# Perforation caused by gastric mucosa associated lymphoid tissue lymphoma: A case report and literature review

**DOI:** 10.1097/MD.0000000000011713

**Published:** 2018-08-17

**Authors:** Xinren Ma, Lei Qin, Yueyu Liu, Wuyang Bian, Ding Sun

**Affiliations:** a The First Affiliated Hospital of Soochow University; b Department of General Surgery, The First Affiliated Hospital of Soochow University, Suzhou, Jiangsu Province, China.

**Keywords:** *Helicobacter pylori* infection, MALT lymphoma, perforation, treatment strategy

## Abstract

**Rationale:**

Gastric mucosa-associated lymphoid tissue (MALT) lymphoma is the most common and best-studied extranodal marginal zone lymphoma of the MALT. It is characterized by an indolent clinical course and excellent survival compared with other malignant tumor. Complications such as obstruction, perforation or bleeding are rarely observed. The treatment strategy is still controversial.

**Patient concerns:**

A 59-year-old man, who had been diagnosed with MALT lymphoma by gastroscopy and biopsy one month before, came to the hospital for a sudden onset of abdominal pain after breakfast.

**Diagnoses:**

MALT lymphoma; gastric perforation.

**Interventions:**

Emergency surgery.

**Outcomes:**

Gastric perforation repair and jejunostomy was performed. The patient recovered well and is preparing for combined chemotherapy.

**Lessons:**

This case report illustrates the challenges in diagnosis and treatment of MALT lymphoma. We discussed the particularity of its clinical characteristics, treatment strategies and prognosis combined with literature review, and we think that early diagnosis and timely appropriate chemotherapy is of great importance.

## 1 Introduction

Gastric mucosa associated lymphoid tissue (MALT) lymphoma is the most common and best-studied extranodal marginal zone lymphoma of the MALT, which accounts for approximately 50% of these diseases. It is characterized by an indolent clinical course and excellent survival compared with the other malignant tumor.^[1]^ The clinical features of gastric MALT lymphoma are nonspecific. It usually manifest as abdominal pain, vomiting, weight loss, etc, which can also be observed in gastric ulcer and any other tumor. Complications such as obstruction, perforation, or bleeding are rarely observed. Herein, we report a case of gastric MALT, which leaded to gastric perforation. Written informed consent was obtained from the patient for publication of this case report and accompanying images.

## 2 Case report

**Figure 1:**
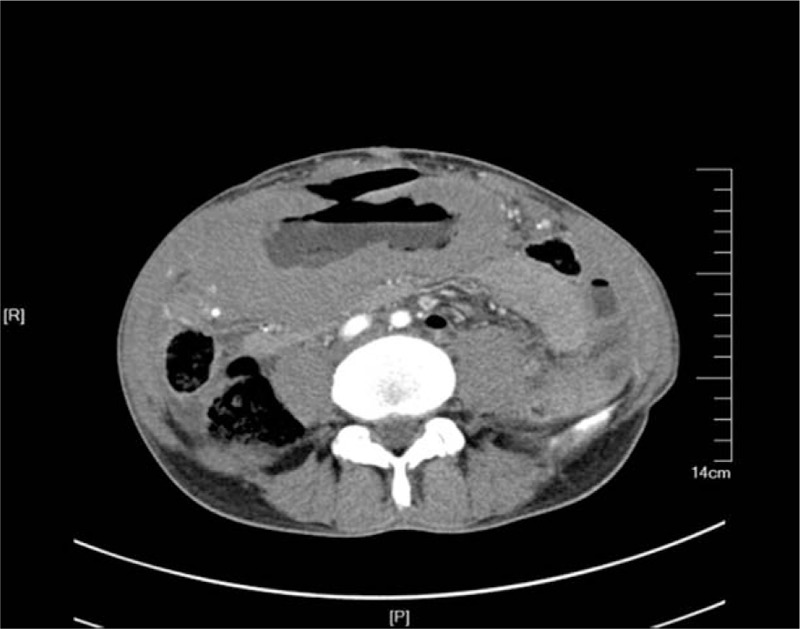
Abdominal CT showed that a crack can be seen on the antetheca of gastric antrum and the gastric wall was diffusely incrassated.

A 59-year-old man was admitted to our department on an emergency basis because of a sudden onset of abdominal pain after breakfast. He was diagnosed with gastric MALT lymphoma 1 month ago but was not given radiation therapy or chemotherapy because of low-level albumin and undernutrition. Abdominal tenderness and tension, rebound pain, and board-like rigidity of abdomen were found throughout physical examination. The abdominal computed tomography (CT) showed that there was free gas and liquid in abdomen. The gastric wall was diffusely incrassated and multiple mass can be seen in the abdomen. There was a crack that can be seen at the antetheca of gastric antrum (Fig. 1).

**Figure 2:**
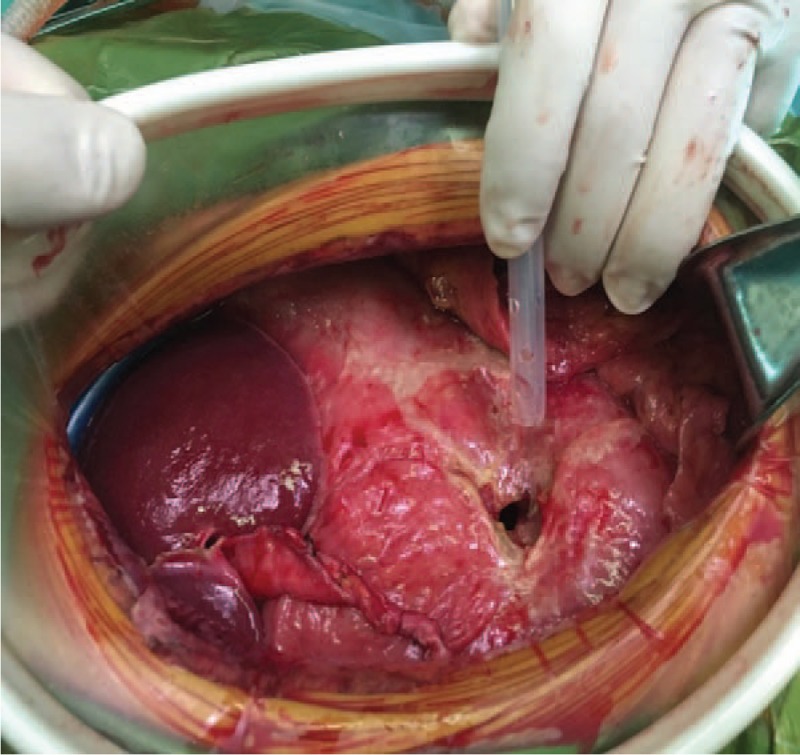
Operation: Gastric perforation (about 2 cm × 2 cm) was found at the gastric antrum during the exploratory laparotomy and gastric perforation repair was performed.

**Figure 3:**
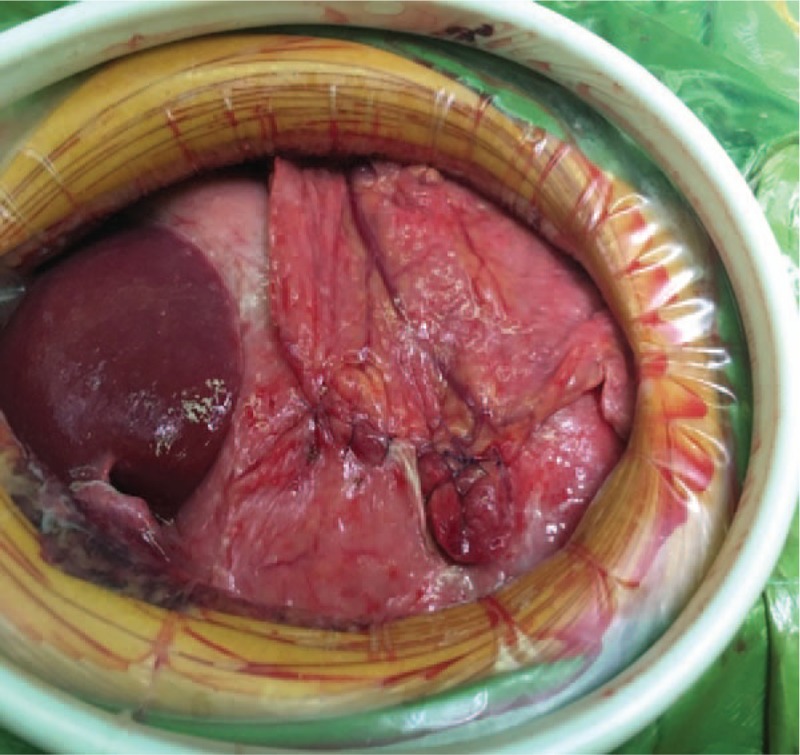
After repair.

The physical and the imaging examination all showed the evidence of gastric perforation, so the exploratory laparotomy was performed right away. There was about 1500 mL yellow-green turbid liquid in abdomen. The gastric wall was thickened and edematous. The lesion, a perforation with a size of about 2 × 2 cm, was found at the anterior gastric wall (Fig. 2). Multiple lymph node metastases were found on the omentum majus, ligamentum hepatogastricum, and retroperitoneum. Luckily, there is no sign of lymph node metastases in thorax. The patient is staged as II_2_E disease according to Musshoff staging system. Considering the surrounding tissue is heavily edematous, gastric perforation repair and jejunostomy was performed. We repaired the perforation with simple interrupted suture (Fig. 3) and covered it with omentum. A fistula with feeding tube was created through the skin at the front of the abdomen and the wall of the jejunum 30 cm away from the ligament of Treitz. The patient was transferred to intensive care unit (ICU) for further treatment.

**Figure 4:**
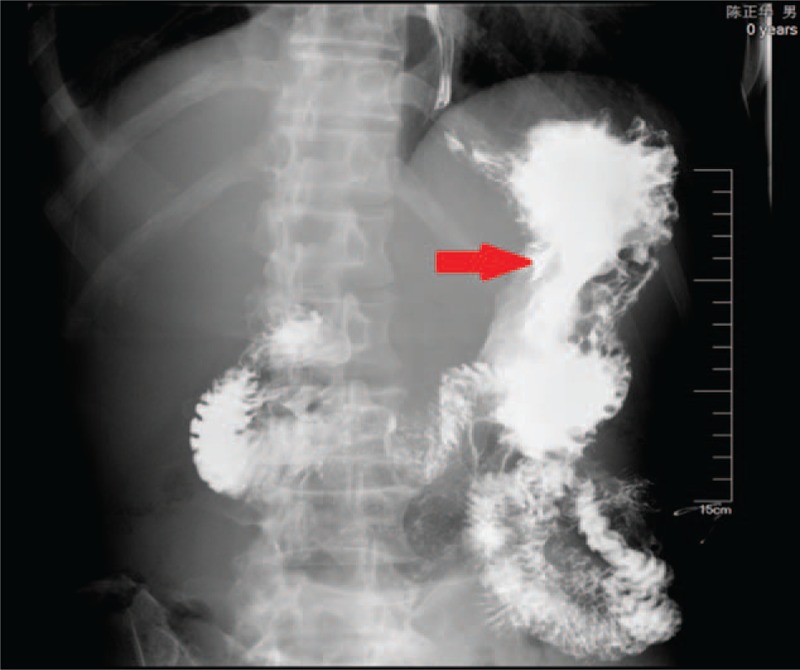
Upper gastroenterography after operation showed the thickening of gastric wall and space-occupying lesion at the gastric antrum.

The patient received parenteral nutrition through jejuna feeding tube 3 days after the operation and was sent back to common ward on the fourth day. Upper gastroenterography after 2 months showed no sign of perforation (Fig. 4). Thickening of gastric wall and space-occupying lesion can be seen at the gastric antrum. The patient is on a liquid diet and will receive formal treatment for lymphoma after general condition improved.

## 3 Discussion

### 3.1 Clinical characteristics

In 1983, Isaacson^[2]^ and Wright introduced the concept of MALT lymphomas, which includes low-grade B-cell lymphoma and immunoproliferative disease. According to the new category, MALT lymphoma refers in particular to the low-grade disease, while the high-grade one is diagnosed with diffuse large B cell lymphoma (DLBCL). Among the numerous organs in which the disease may occur, gastrointestinal tract is the most common one, which accounts for approximately 50%. Meanwhile, 85% of the gastrointestinal MALT lymphomas are the gastric MALT lymphomas.

It is generally acknowledged that gastric MALT lymphoma has a close relationship with *Helicobacter pylori* (*H. pylori*, HP) infection and is influenced by genetic factor, immunosuppressive agent, etc. Evidence shows that chronic HP infection can cause lasting antigenic stimulation, which results in proliferation of lymphocytes. This kind of monoclonal proliferation may evolve into MALT lymphoma.^[3]^ The lesion usually occurs at the pylorus and is localized in mucosa at the early stage. As the disease progresses, the lymphoma may invade muscularis and serosa. When it comes to the advanced stage, it can penetrate the gastric wall, spread to lymph nodes, and cause distant metastasis. The gross manifestations can be characterized by mucosal erosion, thickening of gastric wall, ulceration, and cicatrication. The clinical manifestations are abdominal pain, vomiting, weight loss, etc. These nonspecific symptoms are mimicking chronic gastritis and bring difficulty to early diagnosis. The establishment of diagnosis is based entirely on the histopathological and immunohistochemical examination, which requires a sufficient number of biopsies from both macroscopic lesions and normal appearing mucosa.^[4]^ The reported case is diagnosed with gastric MALT lymphoma by sampling and histopathological findings at an early stage. Due to the inapparent symptoms, lack of attention, and poor nutrition, the patient did not receive formal treatment, which resulted in the progression of the disease and caused penetration in the end. As a matter of fact, complications such as hemorrhage and perforation are rather rare. Perforation may occur in 4% to 10% of MALT lymphoma patients. Mittal et al^[5]^ analyzed the literature up to 1983 included 626 patients and reported that spontaneous perforation occurred in 4% of the patients.

### 3.2 Treatment

The treatment strategy is highly dependent on the clinical stage. Due to the close relationship between gastric MALT lymphoma and HP infection, HP eradication therapy is the first-line treatment of gastric MALT lymphoma, especially for the early-stage one. According to the National Comprehensive Cancer Network (NCCN) guideline, despite the increasing resistance to antibiotics, the regimen including a combination of proton pump inhibitor (PPI) and clarithromycin-based therapy with either amoxicillin or metronidzole for 10 to 14 days is still the first-line therapy of gastric MALT lymphoma.^[6]^ In the reported case, however, the lymphoma had already penetrated the gastric wall and abdominal lymph node metastasis was found in exploratory laparotomy. For these patients, HP eradication therapy is not usually an effective strategy. Some research speculates that at the early stage, the lymphoma is localized in mucosa and its growth relies on HP-specific T-cell mediated immunostimulation. In that case, most of the patients can get complete remission after HP eradication therapy. As the disease progresses, genetic change may occur and the lymphoma cell may become more invasive and invade muscularis and even serosa. At this moment, the lymphoma turns into DLBCL, the growth of which no longer relies on the immunostimulation mediated by T cells and does not respond to HP eradication anymore.^[7]^

As for patients not responding to HP eradication, the strategy is still controversial. Generally speaking, patients with progressive disease or relapse should undergo oncologic treatment, including radiotherapy, chemotherapy, immunotherapy, or a combination of these treatments. The “watch and wait” strategy is also recommended for patients with persistent histologic lymphoma without progressive disease.^[8]^ The lymphoma cells are widely disseminated in gastric wall, and partial gastrectomy often results in relapse so subtotal gastrectomy is not a common strategy for gastric MALT lymphoma. Even though total gastrectomy has the advantage that we can ascertain the pathology and staging of the lymphoma by operation, which is of great importance for further treatment, surgery nowadays plays a less important role in treating gastric MALT lymphoma.^[9]^ As a matter of fact, surgery is restricted to the treatment of complications such as bleeding or perforation. A meta-analysis suggests an equal treatment outcome and a better quality of life with a stomach-preserving therapy after comparing surgical and medical treatment in 5 studies and 700 patients.^[10]^ For patients with advanced-stage lymphoma, systematic chemotherapy is recommended. It is reported that 95% of patients failed in HP eradication therapy, who showed 95% complete remission after combined chlorambucil and rituximab therapy.^[11]^ The reported case is staged as II_2_E disease according to Musshoff staging system. Partial gastrectomy is a lack of efficacy, while total gastrectomy on an emergency basis usually carries a poor prognosis. For the reported case, gastric perforation repair and jejunostomy was performed. After operation, the patient received gastrointestinal decompression. Combined with parenteral nutrition through jejuna feeding tube, most perforation can heal properly. Considering the lymphoma is at progressive stage, we recommend the patient receive combined chemotherapy after general condition is improved.

### 3.3 Prognosis

MALT lymphoma is characterized by an indolent clinical course and excellent survival compared with the other malignant tumor. In a retrospective study by the International Extranodal Lymphoma Study Group (IELSG) of a large series of patients with nongastric MALT lymphoma, the 5-year overall survival (OS) was 90%.^[1]^ The median OS was 12.6 years according to a study that evaluated the survival of patients with marginal zone lymphoma in the unselected population included in the Surveillance, Epidemiology, and End Results (SEERS) database.^[12]^ In contrast to extragastric, gastric MALT lymphomas are presented as localized disease in the majority of cases. The median time to progression is apparently better for the gastric lymphomas compared with the nongastric lymphomas (9 vs 5 years, respectively), but no significant differences in OS have been shown.^[13]^

## Author contributions

**Conceptualization:** Ding Sun.

**Data curation:** Yueyu Liu, Wuyang Bian.

**Formal analysis:** Xinren Ma, Yueyu Liu, Wuyang Bian.

**Investigation:** Lei Qin.

**Methodology:** Ding Sun.

**Project administration:** Ding Sun, Lei Qin.

**Resources:** Lei Qin, Yueyu Liu, Wuyang Bian.

**Software:** Yueyu Liu, Wuyang Bian.

**Supervision:** Lei Qin.

**Validation:** Xinren Ma, Ding Sun, Lei Qin.

**Visualization:** Xinren Ma, Ding Sun.

**Writing – original draft:** Xinren Ma.

**Writing – review & editing:** Xinren Ma, Wuyang Bian.
